# DLC1: a tumor suppressor that regulates Rho signaling

**DOI:** 10.18632/oncotarget.16805

**Published:** 2017-04-04

**Authors:** Brajendra K. Tripathi, Douglas R. Lowy

**Affiliations:** Laboratory of Cellular Oncology, Center for Cancer Research, National Cancer Institute, National Institutes of Health, Bethesda, MD, USA; Laboratory of Cellular Oncology, Center for Cancer Research, National Cancer Institute, National Institutes of Health, Bethesda, MD, USA

**Keywords:** DLC1, Rho-GAP, focal adhesions

Rho GTPases are key transducers of several fundamental cellular processes such as cell division, adhesion, migration, polarity, cytoskeletal remodeling, and apoptosis [3]. They act as molecular switches that cycle between an inactive GDP-bound and an active GTP-bound form in response to intra- or extracellular signaling. This GDP/GTP cycle is carefully controlled by three classes of regulatory proteins: the Rho-GEFs (guanine nucleotide exchange factors) that catalyze the exchange of GDP for GTP, the Rho-GAPs (GTPase activating proteins) that hydrolyze GTP to GDP, and the Rho-GDIs (guanine nucleotide dissociation inhibitors) that form a complex with the GDP-bound form of Rho proteins and prevent nucleotide exchange as well as sequester the Rho GTPases in the cytoplasm. During cell spreading and migration, Rho GTPases control the formation of focal complexes, their maturation to focal adhesions, and the continuous turnover of these structures, along with many aspects of actin cytoskeletal dynamics, including stress fiber formation. Dysregulation of Rho GTPases is associated with diverse abnormal phenotypes and several diseases, including cancer. One Rho regulator that has frequently been implicated in cancer is the tumor suppressor Deleted in Liver Cancer 1 (*DLC1;* also known as *STARD12* or *ARHGAP7*). *DLC1* encodes a Rho-GAP that negatively regulates Rho by converting active GTP-bound Rho to inactive GDP-bound Rho, is associated with focal adhesions, and is down-regulated in many tumor types.

The most abundant translation product of the human *DLC1* gene, which is located at chromosome 8p21-22, is a 1091 amino acid protein that contains three well recognized domains: an N-terminal sterile alpha motif (SAM) domain, a C-terminal steroidogenic acute regulatory protein-related lipid transfer (START) domain, and the Rho-GAP domain, located upstream from the START domain [1, 2, 5]. A Serine-rich Linker region (LR) lies between the SAM and the Rho-GAP domains. A segment within the LR is sometimes referred to as the Focal Adhesion Targeting (FAT) domain because DLC1 is recruited to focal adhesions via this region. The multi-domain structure of DLC1 suggests that it might interact with several other functionally important proteins, and several DLC1 binding partners, in addition to Rho-GTP, have been identified and found to contribute to the biology of DLC1 [1, 5]. Two other structurally related proteins of the DLC family, *DLC2* (STARD13), and *DLC3* (KIAA0189 or STARD8) have also been identified. Although all three members of the family are widely expressed in normal tissues, only DLC1 knockout mice are embryonic lethal, while DLC2 and DLC3 knockout mice are viable, which suggests *DLC1* is essential for embryonic development. In cancer, downregulation of *DLC1* is also more important than *DLC2* and *DLC3* [7]. Fibroblasts cultured from *DLC1*-deficient mouse embryos have shown significant alterations in the cytoskeleton organization of actin filaments and focal adhesions, suggesting a crucial role for DLC1 in these processes in normal cells [2].

DLC1 is a bona fide tumor suppressor that can be down-regulated by gene deletion, point mutation, epigenetic promoter methylation, or post-translational mechanisms in a variety of human cancers, including malignancies of the lung, breast, colon/rectum, prostate, and liver. Restoration of DLC1 expression can inhibits tumor growth and suppresses cell migration *in vivo* and *in vitro*. In addition to its growth suppressing activities, DLC1 has other physiological functions in a variety of normal and transformed cell lines. The most prominent of these is its Rho-GAP activity, which strongly inactivates RhoA, RhoB, and RhoC, weakly inactivates Cdc42, and does not affect Rac1. The catalytic activity of DLC1 is mediated by a conserved “arginine finger,” and mutating this Arginine to Alanine abolishes the Rho-GAP activity of DLC1. Other key functions of DLC1 include its localization to focal adhesions, and the binding of several functionally important proteins such as tensin, which binds to sequences in the LR, and talin and focal adhesion kinase (FAK), which bind to distinct LR sequences located downstream from those that mediate tensin binding. The binding of tensin, talin, and FAK facilitates the association of DLC1 with focal adhesions. During early cell spreading, DLC1 is preferentially localized at the inner/mature focal adhesions, whereas phosphorylated paxillin occupies the outer/nascent focal adhesions. In addition, DLC1 down-regulates paxillin turnover, and does not require its Rho-GAP activity for this function. Instead, it requires the presence of FAK. Both DLC1 and FAK are key regulators of focal adhesion assembly/disassembly that also regulate paxillin turnover at focal adhesions [4].

We have recently determined that direct phosphorylation of four specific Serines in the DLC1 LR by the CDK5 kinase, a serine/threonine kinase, is a common mechanism by which the various functions of DLC1 can be activated [6]. The CDK5-dependent phosphorylations convert the DLC1 protein from a closed inactive conformation to an open active conformation that activates its Rho-GAP activity, facilitates the binding of FAK, tensin and talin proteins to DLC1, and fosters localization of DLC1 to focal adhesions. The findings indicated that, in the absence of these phosphorylations, the LR has an auto-inhibitory function, as it binds tightly to the Rho-GAP domain, resulting in low Rho-GAP activity as well as poor binding of tensin and talin. We have observed this CDK5-dependent mechanism of DLC1 regulation in several non-transformed epithelial cell lines as well as in several cancer lines. These observations, together with other reports [1, 5], suggest that further analyses of post-translational modifications of DLC1 would be worthwhile.
